# Effect on Chest Deformation of Simultaneous Correction of Pectus Excavatum with Scoliosis

**DOI:** 10.1155/2017/8318694

**Published:** 2017-09-12

**Authors:** Jin-Duo Ye, Guang-Pu Lu, Jing-Jing Feng, Wei-Hong Zhong

**Affiliations:** ^1^Tianjin Key Laboratory of the Design and Intelligent Control of the Advanced Mechatronical System, Tianjin University of Technology, Binshuixi Road, No. 391, Tianjin 300384, China; ^2^National Demonstration Center for Experimental Mechanical and Electrical Engineering Education, Tianjin University of Technology, Binshuixi Road, No. 391, Tianjin 300384, China

## Abstract

**Objective:**

This paper is to understand the effect of simultaneous correction of pectus excavatum with scoliosis and to provide some useful information for clinical orthopedic surgery design.

**Methods:**

The method of a three-dimensional reconstruction has been used to the reconstruction of the chest model of pectus excavatum with scoliosis, and the numerical stimulation has been conducted to the process of minimally invasive correction. Three kinds of correction methods have been considered in the numerical simulation, stretch spine, stretch spine and minimally invasive correction at the same time, and release stretch spine after stretch spine and minimally invasive correction of pectus excavatum at the same time.

**Results:**

It is found that stretch spine may help to correction of scoliosis but aggravate the sternum collapse, and release stretch spine after stretch spine and minimally invasive correction at the same time could not only be good at scoliosis but also improve the collapse of the sternum, which could help to improve the heartbeat and breath of the patients.

**Conclusion:**

Among the three kinds of correction methods, release stretch spine after stretch spine and minimally invasive correction at the same time could help to improve both the scoliosis and the collapse of the sternum.

## 1. Introduction

Pectus excavatum (PE) is a common disease with chest wall deformity in adolescents. The incidence in newborns is about 1/300~1/1000, the prevalence rate of male and female is approximately 4 : 1, and some patients may be accompanied with scoliosis [1]. The typical symptom of pectus excavatum was sunken sternum. The symptom can lead to respiratory and circulatory system dysfunction, palpitation, shortness of breath after fatigue, exercise intolerance, and other diseases [2, 3]. The current clinical treatment for pectus excavatum is commonly minimally invasive correction surgery, which replaces the traditional Wada Stern turnover and Ravitch sternal elevation [4]. Its advantages are without osteotomy, shorter operation time, less bleeding, quick recovery, and small and hidden incision [5–7]. The Nuss procedure changed the mechanical environment of the thorax. It also has a pulling effect on the spine which causes the lateral deformation of the spine and is the main cause of postoperative pain. Because of the difference of patients, the operation plan is lack of the support of the numerical results and the Nuss procedure has to rely on the experience of the doctors.

Scoliosis refers to one or several spinal segments deviate from midline bend to side and form a radian. Cobb angle is used to judge the degree of scoliosis deformity. When the angle is 30° < Cobb < 40° [8–10], the patient needs to be treated in hospital. If not promptly treated, it could develop into a serious deformity and will affect the heart and lung function. There are two kinds of treatments of scoliosis, one is nonsurgical treatment and the other is surgical treatment. At present, there are two kinds of effective methods: Boston-type brace [11] and electric stimulation therapy [12]. They are good for the treatment of mild idiopathic scoliosis with Cobb angle less than 15°, which needs no treatment but careful observation. In recent years, there are various methods of surgical treatment; the emergence of new technology, such as Luque method [13], Zieke method, and C-D operation [14, 15], has good effect, but the surgical treatment is easy to injure the spinal cord nerve and cause permanent damage [16, 17]. Pectus excavatum patients are often associated with scoliosis symptoms. According to clinical experience, for patients whose chest wall deformity is not serious, the doctor could perform spine correction and then correct pectus excavatum; a case of right convex of thoracic spine with pectus excavatum was reported in 2005 for the treatment of first spinal traction and then the pectus excavatum correction. For serious pectus excavatum and scoliosis, patients first underwent Nuss procedure and then correction of scoliosis. In 2008, Blane et al. from the University of Michigan first performed a Nuss procedure for a child with scoliosis and then corrected the scoliosis.

At present, there are great progresses for the Nuss procedure research in the field of biomechanics in the world. In 2007, Qiren Zhang from Chang Gung University, Taiwan, made a mechanical analysis of the Nuss minimally invasive correction procedure in three patients with symmetrical funnel chest, but the model did not take into account the spine and did not give the results of stress and strain after correction [18]. In 2010, Nagasao T, a Japanese scholar, created the thorax model by the beam element and compared the postoperative thorax stress distribution between children and adults, in which there were some differences between numerical simulation and clinical results of funnel chest with scoliosis minimally invasive correction process; the numerical simulations predicted the improvement of scoliosis, and scoliosis postoperatively were aggravating [19]. Since 2010, Jin-Duo Ye, from Tianjin University of Technology, has carried out the numerical simulation of the pectus excavatum with scoliosis orthopedic process by using the three-dimensional solid-assembled model and the beam element model. The numerical results of the three-dimensional solid model are consistent with the clinical results. However, in the results of the beam element model, the predicted scoliosis results were significantly different from the clinical results. In 2016, Jin-Duo Ye et al., from Tianjin University of Technology, conducted an experiment on the goat thorax model by using the method of electrical measurement and the strain distribution of the thorax model was obtained [20].

Now, there is less research work on both effects of scoliosis correction on thorax deformation of pectus excavatum and simultaneous correction of pectus excavatum and scoliosis. The author carried out the biomechanics study of simultaneous correction of pectus excavatum and scoliosis by the numerical simulation of minimally invasive correction surgery and scoliosis stretching process and compared the distribution of both the displacement field and stress field of simultaneous correction on thorax deformity.

## 2. Methods

### 2.1. Construction of Finite Element Model

**Figure 1:**
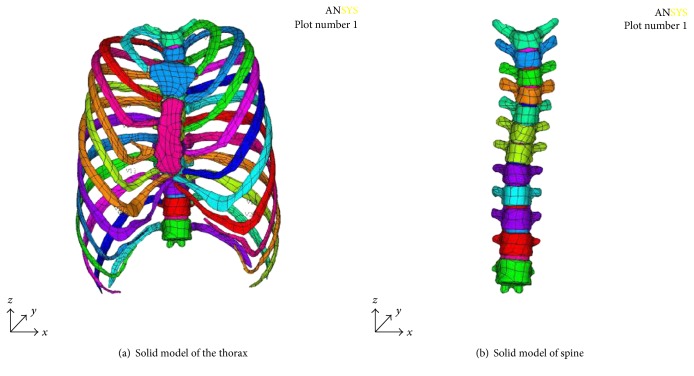
3-D solid model of PE with scoliosis.

Computed tomography (CT) images of thorax of the patient with pectus excavatum with scoliosis were provided by General Hospital of Beijing Command. In the patient with pectus excavatum with scoliosis, the sternum concaves inward to the right side with a depth of 40.3 mm, and the spine bows to the left with a Cobb angle of 20°. The 3-D solid model of the deformed thorax was reconstructed in Mimics. The model was imported into Geomagic for point cloud processing and finally imported into ANSYS to create the assembled solid model (Figure 1). Figure 1 was the thorax model with discs. It included two pieces of sternums, twelve pairs of ribs, and twelve section of thoracic vertebras, and the coordinates of the model are in accordance with the previous CT default coordinate system. In the model, the method of coupling displacement has been used to link the vertebras and the ribs and the ribs and the sternum. The intervertebral discs are obtained by Boolean operation between two vertebrae. Finally, the 3-D solid model of the thorax is shown in Figures 1(a) and 1(b).

For the pretreatment of the model, element type is set solid45 and material parameters are set linear isotropic. The elastic modulus of sternum, thoracic vertebrae, and ribs is set to E = 380 MPa, Poisson's ratio is *μ* = 0.3, the elastic modulus of the intervertebral disc is set to E = 10 MPa, and Poisson's ratio is *μ* = 0.45 [21].

**Figure 2:**
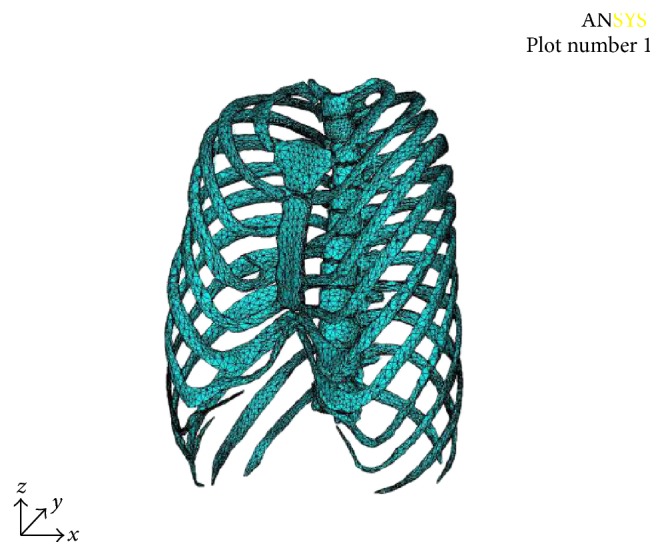
Finite element model.

The mesh is divided into 106,913 elements and 56,584 nodes. The finite element model is shown in Figure 2.

### 2.2. Correction of Scoliosis

#### 2.2.1. Boundary Conditions

The lower surface of T_12_ of thoracic vertebrae is fully constrained, and the 11 mm displacement along the *z*-axis is applied to the upper surface of T_1_ to simulate the correction of spine. The connection of clavicle and the manubrium sterni has been taken into account, and the influence of the restraint of the sternum on the deformation of the chest has been also taken into account in the paper. Considering the geometric nonlinearity, we set the appropriate load step, adopt the residual force convergence criterion, set the convergence precision to 0.001.

### 2.3. Simultaneous Correction of Pectus Excavatum with Scoliosis

#### 2.3.1. Boundary Condition

In this section, the numerical simulation sets two load steps. In the first step, the T_12_ thorax bottom surface displacement is constrained. Tensile displacement along the axial is 8 mm on the upper thorax spine T_1_, which was to simulate the stretching of the spine. Minimally invasive orthopedic correction displacement of pectus excavatum applying on the second sternum is −40 mm of Y direction. In the second load step, the axial displacement of the T_1_ segment of the thorax spine was released and the displacement of Y orientation was kept. Because of the large deformation of the ribs in the process of correction, the geometric nonlinearity has been considered. The last step is the option of nonlinear solution, such as step length, step number, calculation time, and calculation results of output frequency, and precision of residual force convergence criterion is set to 0.001.

## 3. Results

### 3.1. Spine Correction Displacement Analysis

**Figure 3:**
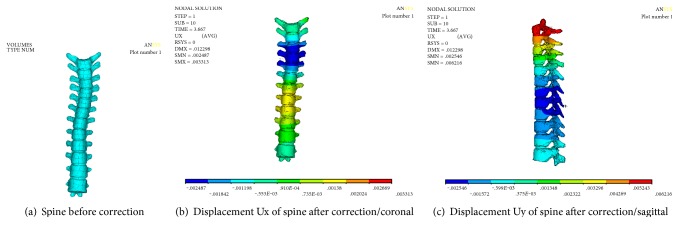
Displacement of spine.

Numerical results of displacement are shown in Figure 3.  Figure 3(a) is the spine before correction; it shows that the thoracic vertebra T_3_-T_4_ segment bends to the right (X positive direction, coronal plane), thoracic vertebra T_7_–T_9_ segment bends to the left (X negative direction, coronal plane). From Figure 3(b), after correction, the maximum displacement of Ux is 3.313 mm in thoracic vertebra T_7–_T_9_, moved to left; the minimum displacement of Ux is −2.487 mm in thoracic vertebra T_3_-T_4_, moved to right. The results show that the spine has been corrected.

### 3.2. The Analysis of Thorax Displacement

**Figure 4:**
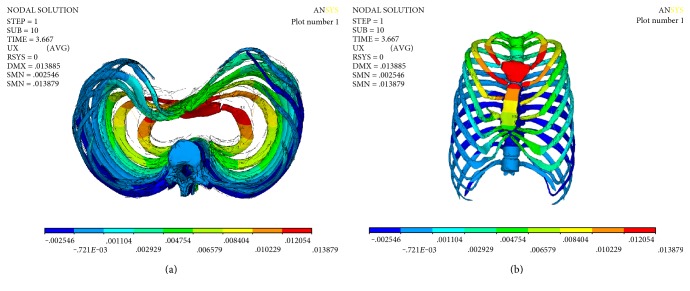
Displacement Uy of thorax ((a) cross, (b) coronal).

**Figure 5:**
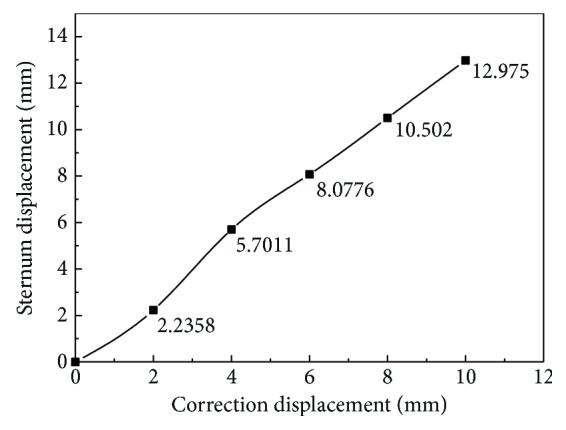
Displacement vector of thorax.

The numerical results of the displacement are shown in Figure 4(). It can be seen from Figure 4() (cross section) that the sternum depression is more serious after scoliosis correction, and the maximum value of displacement located on the first rib which is connected to the sternum is 13.879 mm. The displacement of the manubrium sterni is larger than that of sternum. The relationship between the displacement of the sternum and the correction displacement is plotted in Figure 5(). Figure 5() shows the spine stretch displacement is less than 2 mm; the displacement of the sternum is smaller; after 2 mm, the subsidence displacement of the sternum increased rapidly; and when the displacement is more than 4 mm, there is a linear relationship between the displacement of the sternum and the spine stretch displacement.

**Figure 6:**
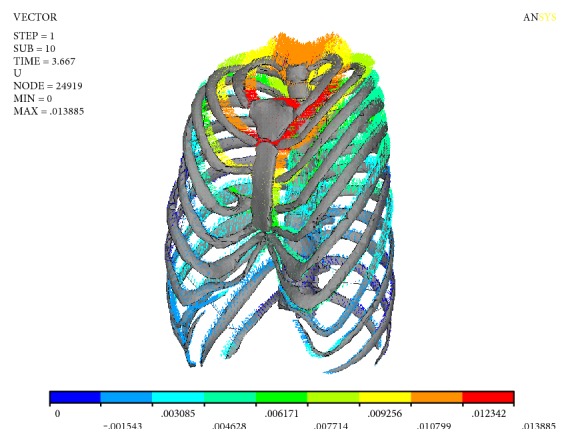
Displacement curve of sternum.

Figure 6 shows the deformation vector of the thorax. It can be seen from Figure 6 that the displacements of the sternum, the first rib, the second rib, and the thoracic vertebra T_1_-T_2_ segment are larger than those of others. In the process of stretching the spine, not only the scoliosis of the spine was corrected but also the normal physiological kyphosis of the spine was changed. It can be seen from Figure 3(c) that the displacement of T_1_-T_2_ segment of thoracic vertebra is the largest in sagittal plane, and the maximum value is 6.216 mm. The first pair of ribs and the second pair of ribs connect to thoracic vertebra T_1_-T_2_, manubrium sterni, and sternum; the function of the ribs is like a rod, so when the thoracic vertebrae T_1_-T_2_ segment move in the sagittal plane, it will drive the displacement of manubrium sterni and sternum and result in a further collapse of the thorax. The numerical results clearly explain the phenomenon of hypotension in patients; it is the correction of scoliosis that effected the breathing and heartbeat [22].

The numerical simulation results show that the displacement boundary condition at the connection of the clavicle and the manubrium sterni has a significant influence on the deformation of the thorax. The influence of the boundary condition both in X direction (coronal plane) and in the axial direction of spine has relatively less influence on the deformation of the chest, but the boundary condition in sagittal plane has a great influence on the deformation of the chest. Once the sagittal displacement of the sternum is completely restrained, the sternum is no longer collapsing to the spine, which is not consistent with the appearance of hypotension in the patients of pectus excavatum with scoliosis treated in the literature [22].

### 3.3. Stress Analysis of Correction of Spine

**Figure 7:**
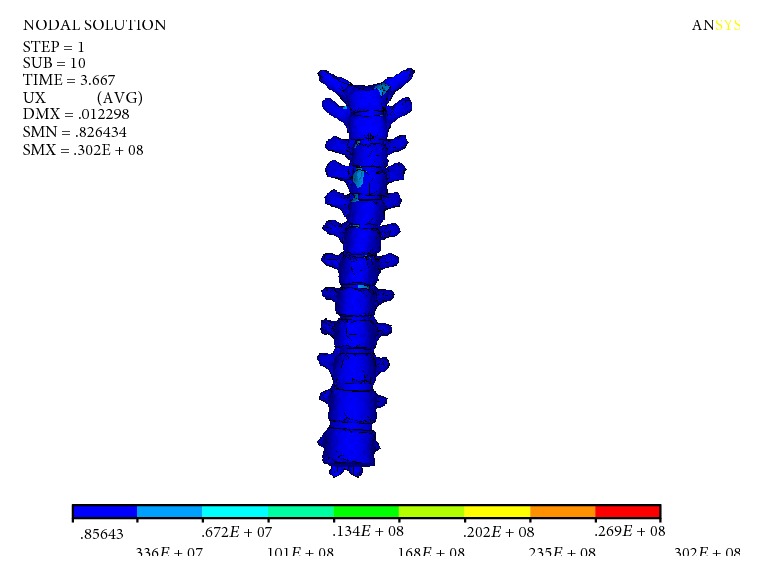
Mises stress of spine.

**Figure 8:**
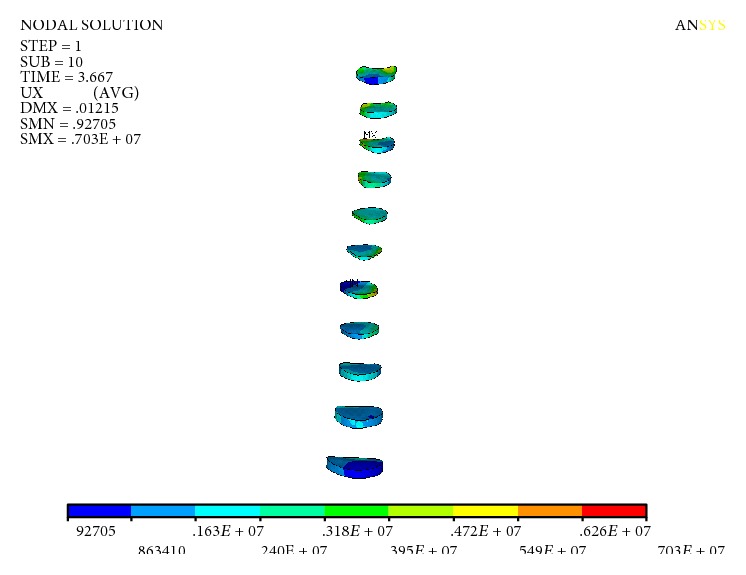
Mises stress of intervertebral discs.

The Mises stress of the spine is shown in Figure 7. The maximum stress of the spine was 30.2 MPa, which appeared in the thoracic vertebra T_1_-T_2_ segment. The rest of the stress is smaller. The Mises stress of the intervertebral disc is shown in Figure 8, and the maximum stress of the intervertebral disc is 7.03 MPa.

### 3.4. Analysis of Correction Force of Spine

**Figure 9:**
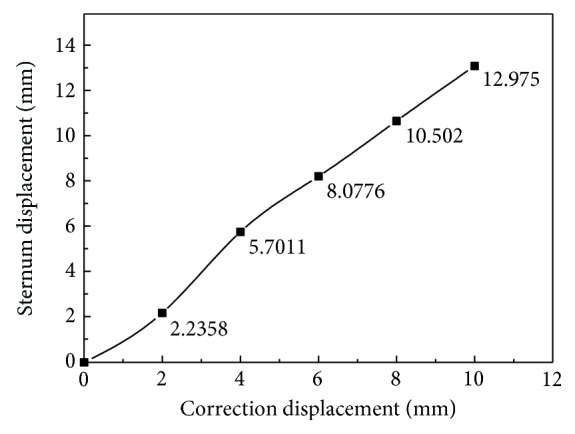
Curve of correction force displacement.

The numerical results of the correction force are shown in Figure 9(); correction force is proportional to correction displacement; with the increase of correction displacement, the correction force gradually increased. When the correction is completed, the maximum correction force reaches 467.9 N. With the increase of displacement correction, Cobb angle decreases, correction torque arm decreases, and correction torque depends on the correction force and correction arm. When the lateral bending angle decreases, the correction force increases.

### 3.5. Numerical Simulation Results of Nuss Procedure and Stretch of Spine

The displacement and stress of the thorax can be obtained through numerical simulation of minimally invasive surgery and stretch of spine. Figures 0() and 1() show the displacement of the chest in sagittal and coronal plane. It can be seen that the sternum (sagittal plane) was raised to 41.953 mm, followed by the upward movement of the ribs. The collapse symptom of pectus excavatum was improved. The maximum value Ux of the displacement on the coronal plane is 2.19 mm, which is located on the T_7_-T_8_ segment of the spine. The minimum value is −2.995 mm, which is located on the T_3_-T_4_ segment of the spine. The maximum displacement of the spine is 8.176 mm; in the direction of axis of spine, the Cobb angle was significantly decreased. The numerical results have achieved the effect of simultaneous correction of pectus excavatum and scoliosis. The Mises stress is uniform; the maximum Mises stress is located on the sternum and thoracic vertebras at T_1_–T_4_, and the maximum values were 76.6 MPa and 23.4 MPa, respectively. It may be the main reason of the pain of Nuss procedure and the correction of spine.

**Figure 12:**
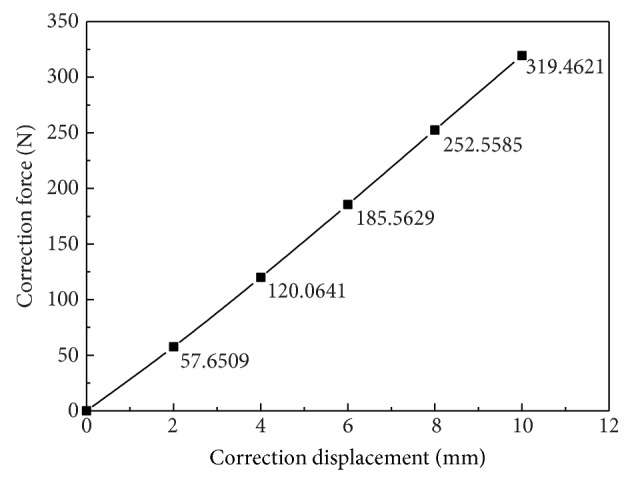
Correction force-displacement curve.

The correction force displacement of the numerical calculation is shown in Figure 12. The relation of correction force and the displacement of the correction are basically linear. The correction force is to increase along with the increase of the displacement. When the correction is completed, the maximum correction force reaches 319.52 N. At the beginning of correction, the correction force is small. With the increase of the displacement correction, the symptoms of scoliosis decreased, Cobb angle decreased, and correction force increased.

### 3.6. Numerical Simulation Results after Releasing of Stretch Correction

**Figure 13:**
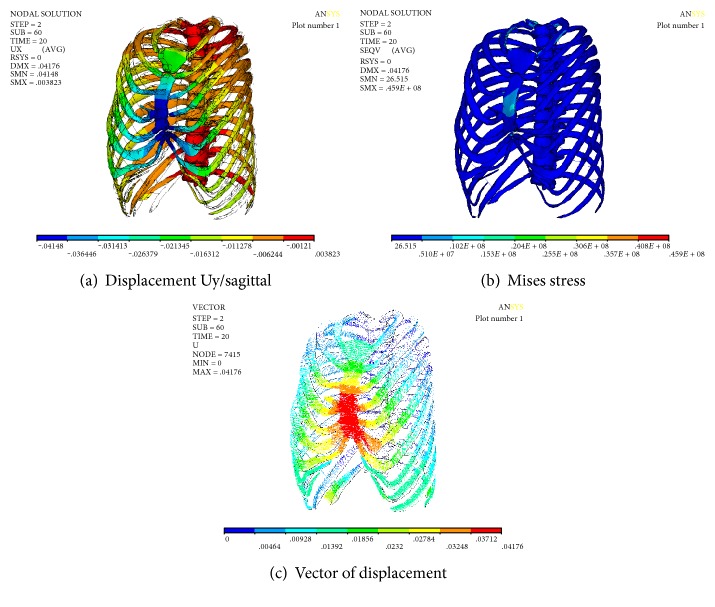
Numerical results of the release stretch of spine.

The axial displacement of the spine is removed, and only the minimally invasive correction displacement of pectus excavatum is kept. The displacement of the thorax on sagittal plane is shown in Figure 13(a); the numerical result shows that the maximum displacement of correction is −41.48 mm, occurred on the lower piece of the sternums, and the ribs of left four, left five, and left six and right three, right four, right five, and right six are significantly raised. The overall funnel chest symptoms improved significantly.  Figure 13(b) shows Mises stress distribution of thorax after correction; we can see that the maximum stress position is on the sternum, which is consistent with the position of correction displacement. The maximum stress is 45.9 MPa.

Figure 13(c) shows the chest vector of displacement; it can be seen that the greater displacement is located on the sternum. In addition to the correction of the lateral curvature of the spine, it also corrects the normal convex in the process of stretching the spine.

**Figure 14:**
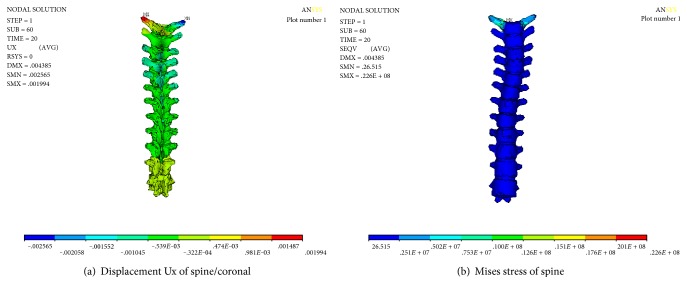
Numerical results of spine after stretching and releasing.

The numerical results of correction and Mises stress of the spine are shown in Figure 14; from the numerical results, it can be seen that T_3_–T_5_ section of the thoracic spine on both sides of the transverse moves around 1 mm to the left (X direction), it indicates that the funnel chest and the spinal column side bending simultaneously could help to improve the scoliosis and that the maximum stress of the spine is 22.6 MPa, which is located in the cross section of the T_1_ segment of the thoracic spine. This may be related to the load way of displacement.

**Figure 15:**
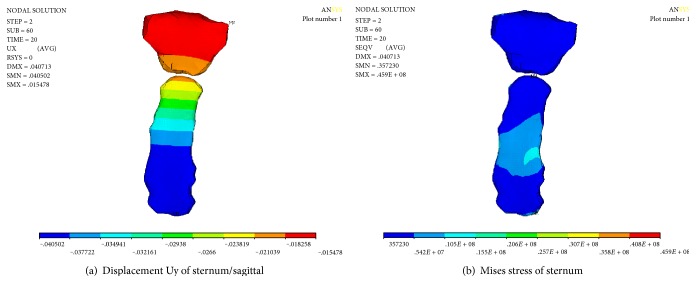
Numerical results of sternum after stretching and releasing.

As shown in Figure 15, the displacement change of the sternum is gradient. The maximum displacement is located on the sternum. The maximum value is 40.502 mm. The minimum displacement of the upper manubrium sterni is 15.478 mm. The maximum stress of the sternum is 45.9 MPa.

**Figure 16:**
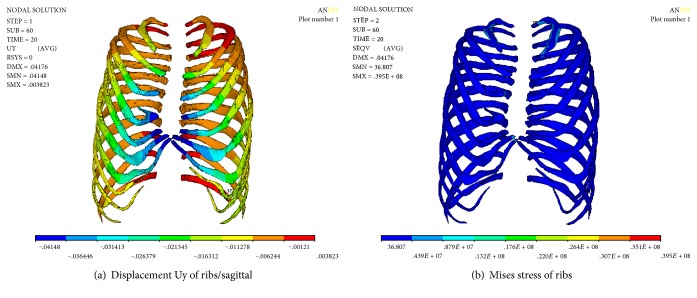
Numerical results of ribs after stretching and releasing.

Figure 16 shows the displacement and the Mises stress distribution along the sagittal plane after correction. A more serious collapse occurred on the ribs of left four and left five, and at same time, the ribs of right four and right five were significantly elevated; funnel chest symptom was improved. The maximum stress is 39.5 MPa, which is located on the right side of the rib. It may be the stress concentration caused by the load way of spine correction.

### 3.7. Comparison of Numerical Results of the Correction of Pectus Excavatum with Scoliosis

As seen from Table 1, there is less difference of the maximum displacements of the sternum except the stretching correction and there is much difference of the minimum displacements of the manubrium sterni. According to the results of removing the displacement of stretch spine, the result of release of the stretch spine is better than that of the Nuss procedure. The results show that simultaneous correction and release of the spine stretch is beneficial to the improvement of pectus excavatum. Meanwhile, the maximum stress of releasing stretch scoliosis is close to that of the Nuss procedure.

## 4. Discussion

There are many factors influencing on the deformation of both thorax and spine. In this paper, we investigated the effect of stretching of the spine on the deformation of the chest from the biomechanical point of view. So far, there have been no definite conclusions about the correction order of pectus excavatum and scoliosis. Pectus excavatum patients are often associated with scoliosis symptoms. According to clinical experience, for patients whose chest wall deformity is not serious, the doctor could perform spine correction and then correct pectus excavatum; Huizhong Tian reported a case of right convex of thoracic spine with pectus excavatum for the treatment of first spinal traction and then the pectus excavatum correction in 2005, which achieved a satisfactory result [23]. However, for the patients with serious pectus excavatum and scoliosis, the treatment should first be the Nuss procedures and then scoliosis correction. In 2008, Blane et al. from the University of Michigan first performed a Nuss procedure for a child of pectus excavatum with scoliosis and then corrected the scoliosis [22]. The deformity of thorax in funnel chest with scoliosis is extremely complicated and varied. Many factors such as the location and depth of the funnel chest and the lateral bending of the spine may affect the correction outcome. The purpose of this paper is to explore a surgical approach to reduce the risk of pain in patients and to improve the symptoms of funnel chest and scoliosis, providing a reference for the design of clinical surgery.

## 5. Conclusion

1. In this paper, numerical simulation method was used to simulate the process of the Nuss procedure and scoliosis-stretching correction simultaneously. It is found that stretch of spine did influence the deformation and stress of the thorax. The numerical results clearly explain the phenomenon of hypotension in patients; it is the correction of scoliosis that effected the breathing and heartbeat.
2. Numerical simulation results have shown that correction of both pectus excavatum and scoliosis simultaneously could help to improve of the symptom of both pectus excavatum and scoliosis. At the same time, releasing of stretch displacement after correction of spine could help to raise the sternum and is favorable to the Nuss procedure.
3. Comparing different correction method in the paper, it is found that the displacement of manubrium sterni by releasing stretch of spine was greater than that of the Nuss procedure and the Mises stress of the sternum is close to that of the Nuss procedure. Comparing the results of simultaneous correction, the results of the Nuss procedure, and the results of released correction of stretch spine, it is found that the loading path affects the correction results, which suggests that not only the process of Nuss procedure but also the process of the stretch of spine are nonlinear rather than linear.

## Figures and Tables

**Figure 10:**
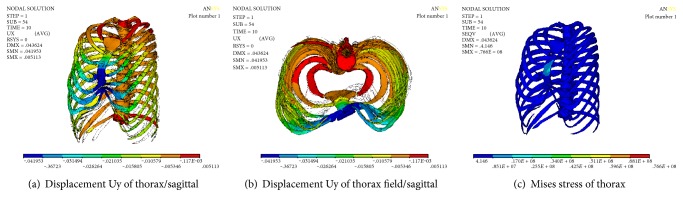
Numerical results of simultaneous correction of PE with scoliosis.

**Figure 11:**
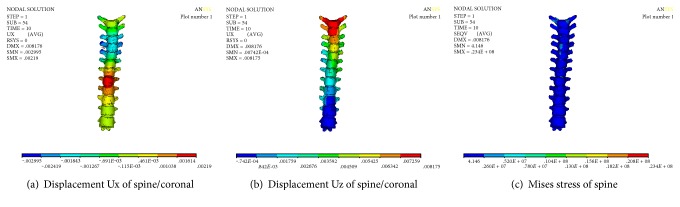
Numerical results of spine/simultaneous correction of PE with scoliosis.

**Table 1:** Comparison of the numerical results.

Method of correction	Maximum displacement/sternum (/mm)	Minimum displacement/manubrium sterni (/mm)	Mises stress of sternum (/MPa)
Simultaneous correction	40.502	15.478	45.9
Released correction of stretch spine	40.743	2.120	70.5
Stretch spine	−25.460	−13.879	30.2
Nuss procedure	40.737	0.210	70.3

## Abbreviations

PE: Pectus excavatum
CT: Computed tomography
DICOM: Digital imagine and communication in medicine
DOF: Degree of freedom.
